# Effect of Magnesium Supplementation on the Distribution Patterns of Zinc, Copper, and Magnesium in Rabbits Exposed to Prolonged Cadmium Intoxication

**DOI:** 10.1100/2012/572514

**Published:** 2012-06-04

**Authors:** Zorica Bulat, Danijela Đukić-Ćosić, Biljana Antonijević, Petar Bulat, Dragana Vujanović, Aleksandra Buha, Vesna Matović

**Affiliations:** ^1^Department of Toxicology “Akademik Danilo Soldatović”, Faculty of Pharmacy, University of Belgrade, Vojvode Stepe 450, 11221 Belgrade, Serbia; ^2^Institute of Occupational Health, Faculty of Medicine, University of Belgrade, Deligradska 29, 11000 Belgrade, Serbia

## Abstract

The present study is designed to investigate whether magnesium (Mg) supplementation may prevent Cd-induced alterations in zinc (Zn), copper (Cu), and magnesium (Mg) status in rabbits. For this purpose, the concentrations of Zn, Cu, and Mg were estimated in blood, urine, and organs (brain, heart, lungs, liver, kidney, spleen, pancreas, skeletal muscle, and bone) of rabbits given Cd (10 mg/kg b.w.) and rabbits cotreated with Mg (40 mg/kg b.w.) orally, as aqueous solutions of Cd chloride and Mg acetate every day for 4 weeks. Samples were mineralized with conc. HNO_3_ and HClO_4_ (4:1) and metals concentrations were determined by atomic absorption spectrophotometry (AAS). Magnesium supplementation succeeded to overcome Cd-induced disbalance of investigated bioelements. Beneficial effects of Mg were observed on Zn levels in blood and urine, on Cu levels in urine, and on Mg levels in blood. Magnesium pretreatment also managed to counteract or reduce all Cd-induced changes in levels of Cu and Mg in organs, while it did not exert this effect on Zn levels. These findings suggest that enhanced dietary Mg intake during Cd exposure can have at least partly beneficial effect on Cd-induced alterations in homeostasis of zinc, copper, and magnesium.

## 1. Introduction

Over the past several decades, numerous experimental and epidemiological studies on cadmium (Cd), as an important environmental and occupational toxicant, demonstrated multiple mechanisms of Cd toxicity. Besides mechanisms of Cd toxicity which include induction of oxidative stress and apoptosis, aberrant gene expression, altered DNA structure, and inhibition of ATP production in mitochondria [1–4], Cd toxicity can be also explained by disturbed homeostasis of bioelements [5–7]. The increasing environmental cadmium exposure, on one hand, and the wide-spread bioelements deficiency in the world mainly due to nutritional factors but also as a result of cadmium exposure, on the other hand, clearly indicate the relevance of Cd bioelements interactions [8–11].

Numerous studies provided evidence that supplementation with certain essential elements, especially zinc (Zn) and selenium (Se), can have protective role against Cd toxicity [6, 7, 12, 13]. The literature data indicate that enhanced Zn intake reduced Cd body burden [14, 15], had beneficial effect on lipid peroxidation induced by Cd [13] or skeletal properties impaired by Cd [16], and had even protective effect against Cd-induced carcinogenicity [4]. Supplementation with Se reduces Cd concentration [17] and protects against Cd-induced oxidative stress in various organs [18]. Although investigated in less extent, there are evidences on beneficial effect of magnesium (Mg) supplemental intake on Cd toxicity. Magnesium reduced Cd concentration and lipid peroxidation and had even protective effect against carcinogenicity and teratogenicity of Cd in experimental animals as reviewed by Matović et al. [2, 19]. Our recent studies have shown that supplementation with Mg significantly reduces Cd concentration in the blood, kidney, spleen, and bone of rabbits exposed to prolonged Cd intoxication [15] and has beneficial effect on Cd levels in kidney, lungs, testis, and spleen, as well as kidney glutathione (GSH) concentration in mice exposed to subacute intoxication [20–22]. However, data on Mg effect on the fate of essential metals that are disturbed in conditions of Cd intoxication are very limited. Thus, the aim of this study was to investigate the effect of Mg supplementation on the level of Mg, Zn, and copper (Cu) in rabbits exposed to prolonged Cd intoxication.

## 2. Materials and Methods

### 2.1. Chemicals

All reagents and chemicals used were of analytical grade quality or higher purity. Cadmium chloride (CdCl_2_·H_2_O), magnesium acetate [Mg(CH_3_COO)_2_·2H_2_O], trace-pure concentrated nitric and perchloric acids, and metals standard solutions for atomic absorption spectrometry (AAS) were purchased from Merck (Darmstadt, Germany). Double-distilled water was used in metals analysis.

### 2.2. Animals and Experimental Protocol

The experiment was performed on rabbits *Oryctolagus cuniculus-Belgian hare*, weighting 2.5-3.5 kg. Throughout the experiment, the animals were maintained in accordance with institutional and international guidelines (European Community Guidelines). The experimental protocol was approved by the Ethics Committee of the Military Medical Academy, Belgrade, Serbia.

Animals were kept under controlled conventional conditions (temperature 22 ± 2°C, relative humidity of 50 ± 10%, 12 h light-dark cycle) and were housed individually in standard cages. They had free access to drinking water and standard pellet diet which contained minimum 16% protein, maximum 12% cellulose, minimum 1.0% Ca, minimum 0.8% P, minimum 50 mg Zn/kg, and minimum 8 mg Cu/kg (manufacture's data). The following concentrations of metals were determined in our laboratory: 91 mg Zn/kg, 21 mg Cu/kg, 2.4 g Mg/kg, and 19.2 *μ*g Cd/kg in diet and in drinking water 148 *μ*g Zn/L, 10 *μ*g Cu/L, and 15 mg Mg/L, while Cd concentration was under 0.1 *μ*g/L.

The rabbits were randomly divided into 3 groups containing eight animals each:

- *Control*: nontreated animals;
- *Cd group*: rabbits given orally, by orogastric tube, every day for 4 weeks 10 mg Cd/kg b.w. as aqueous solution of CdCl_2_;
- *Cd + Mg group*: rabbits exposed to the same dose of Cd and, 1 h later, supplemented orally with 40 mg Mg/kg b.w. as aqueous solution of Mg(CH_3_COO)_2_.

Before and during intoxication (0, 10th, 14th, 18th, 22nd, 25th, and 28th day), blood samples were taken from the ear arteries using a cannula and collected in tubes with sodium heparin as anticoagulant.

The rabbits were housed individually in metabolic cages immediately after Cd or Cd + Mg application and were provided with water only, and 24-hour urine samples were collected at days 0, 10, 15, 17, 19, 21, 23, 25, and 28 of investigation.

At the end of experiment (28th day), all animals were sacrificed by injection of 3 mL of a 50 g/L sodium pentobarbitone solution in the marginal vein of the ear which was followed by ear emboli. Nine organs (brain, heart, lungs, kidney, liver, spleen, pancreas, skeletal muscle, and bone) were excised and stored frozen (−20°C) until analysis. Left cerebral hemisphere, left kidney, dorsocaudal part of the heart, proximal part of the left femoral bone (without bone marrow) and muscle, part of the liver (from lobus hepatis sinister), part of the left lung (from lobus caudalis), and whole spleen and pancreas were used for further analysis.

### 2.3. Sample Preparation and Analytical Method

Samples of whole blood, urine (filtered and evaporated to small volume), and soft tissues were mineralized with concentrated or conc. HNO_3_ and HClO_4_ in 4/1 ratio. After mineralization, samples were diluted with 0.1 mol/L HNO_3_, and the concentrations of metals were determined by flame atomic absorption spectrophotometry (FAAS, instrument GBC 932AA, Dandenong, Australia). The accuracy of the AAS analyses was validated with reference samples from the National Bureau of Standards (NIST SRM 1577a bovine liver, National Institute of Standards and Technology, Gaithersburg, MD, USA).

### 2.4. Statistical Analyses

Statistical analyses of results were conducted by one-way analysis of variance (ANOVA) followed by the LSD multiple comparison test for metals concentrations in blood and organs, as well as for bioelements ratios in blood, liver, and kidney. Because of the skewed distribution of determined levels of bioelements in urine, the significance of difference between groups was calculated by Mann-Whitney test. Pearson's rank correlation was conducted to investigate relationship between Zn, Cu, and Mg concentrations in blood, liver, and kidney.

All values are presented as means ± SD. The acceptable level of significance was set in all cases at *P* < 0.05. All calculations were prepared with EXCEL 2007 and SPSS package PASW Statistics 18.

## 3. Results

### 3.1. Zn, Cu, and Mg Concentration in Blood of Rabbits Exposed to Cd and Cd + Mg

**Figure 1:**
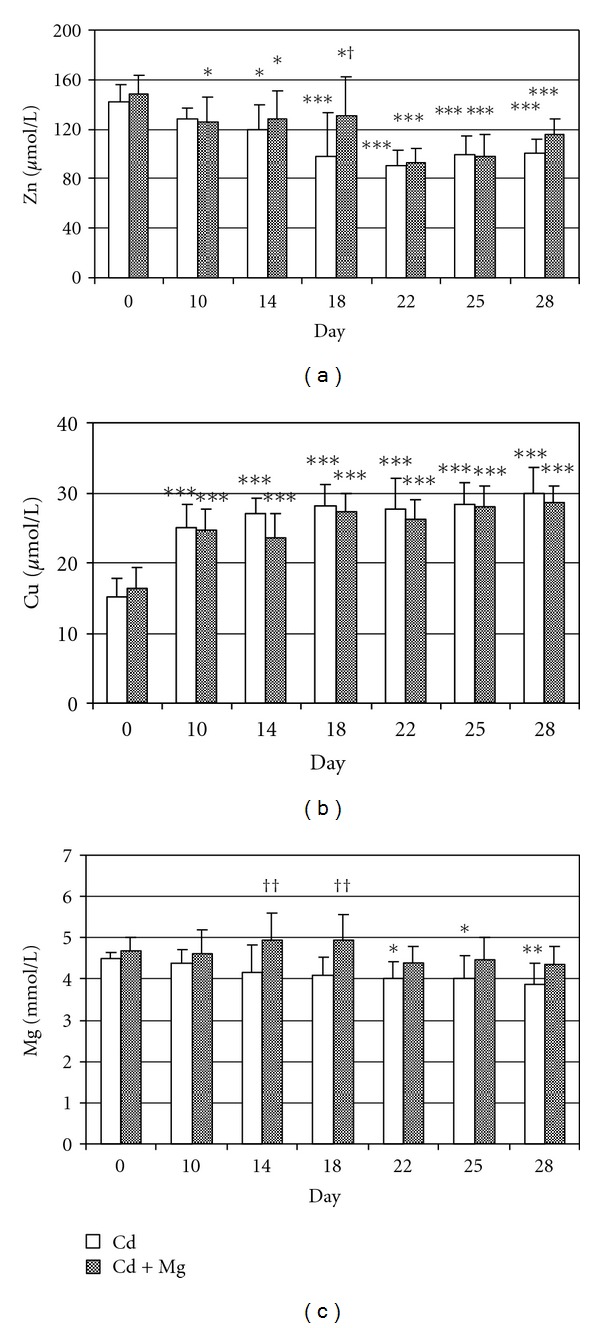
The effect of Mg supplementation on Zn (a), Cu (b), and Mg (c) concentration in the blood of rabbits intoxicated with Cd. Cd group intoxicated orally for 4 weeks with 10 mg Cd/kg b.w./day. Cd + Mg group given 40 mg Mg/kg b.w. 1 h after Cd treatment. Marked values differ significantly (ANOVA + LSD test) from *control (day 0) and ^†^Cd group (*P* < 0.05). ^∗, †^ *P* < 0.05; ^∗∗, ††^ *P* < 0.01; ***P < 0.001.

Both Cd and Cd + Mg treatment induced decrease in blood Zn concentration, which was statistically significant from the 14th or 10th day, respectively, until the end of experiment when the blood Zn concentration was 20–30% lower than at zero time (Figure 1(a)). However, Mg cotreatment succeeded to significantly elevate Zn level in blood on 18th day, compared with Cd group.

Contrary to Zn, blood Cu levels were in both groups profoundly increased (up to 50%) during the entire experiment compared with values determined on the day 0 (*P* < 0.001), and no significant difference between the groups was observed (Figure 1(b)).

Cadmium intoxication induced significant decrease of blood Mg levels from the 22nd day till the end of experiment (Figure 1(c)). Magnesium administration managed to prevent Cd-induced changes in blood Mg concentrations that were significantly higher (*P* < 0.01) at days 14th and 18th if compared to Cd-intoxicated animals. Moreover, no significant changes were obtained in blood of rabbits cotreated with Mg if compared with control value (day 0).

### 3.2. Zn, Cu, and Mg Concentration in Urine of Rabbits Exposed to Cd and Cd + Mg

Elimination of Zn, Cu, and Mg via urine of rabbits treated with Cd or Cd + Mg is presented in Table 1.

At the beginning of the experiment, the mean concentrations of Zn in urine were around 10 *μ*mol/L. Cadmium induced marked increase in Zn elimination via urine on the 21st and 23rd day compared with day 0, when Zn concentration reached values higher than 20 *μ*mol/L. On the other hand, magnesium supplementation resulted in nearly unchanged urine Zn concentration compared to Zn concentrations in urine of Cd group during the entire experiment.

A two-fold increase of Cu elimination via urine was registered in animals intoxicated with Cd from the third week until the end of experiment, while Mg administration resulted in nearly unchanged Cu concentration in urine if compared with control values (day 0). When compared with Cd group, Mg cotreatment significantly decreased Cu urine concentration on the 21st day.

Enhanced Mg elimination via urine observed in Cd-intoxicated animals was potentiated by Mg supplementation with urinary Mg concentration almost 4 times increased after 17 and 19 days if compared with 0 time (*P* < 0.001). Although Mg treatment induced statistically significant enhancement of Mg elimination via urine in nearly all investigated intervals (if compared with Cd group), no significance between Cd and Cd + Mg group was observed at the end of the experiment.

### 3.3. Zn, Cu, and Mg Concentration in Organs of Rabbits Exposed to Cd and Cd + Mg

Cd intoxication, as well as Mg cotreatment, induced significant increase of Zn concentration in liver and spleen (*P* < 0.01) if compared with control levels of Zn, while a decrease was observed in bones (Table 2). However, no alterations of Zn concentration in investigated organs were observed between Cd and Cd + Mg groups after 4 weeks of intoxication.

Copper concentration was significantly increased in kidney, muscle, pancreas, and spleen of animals exposed to Cd in comparison to the control group. Nevertheless, cotreatment with Mg had beneficial effect on Cu concentration in these organs in which Cu levels were within the range of control. Consequently, Cu levels were significantly lower in kidney (*P* < 0.001), brain (*P* < 0.01), and muscle in Cd + Mg group if compared with Cd group.

Cadmium intoxication decreased Mg concentration in muscles and increased its concentration in spleen. Supplemental Mg counteracted these alterations since no differences between Cd + Mg and control group were observed in investigated organs.

### 3.4. Zn, Cu, and Mg Ratios and Their Correlations in Blood, Liver, and Kidney of Rabbits Exposed to Cd and Cd + Mg

**Figure 2:**
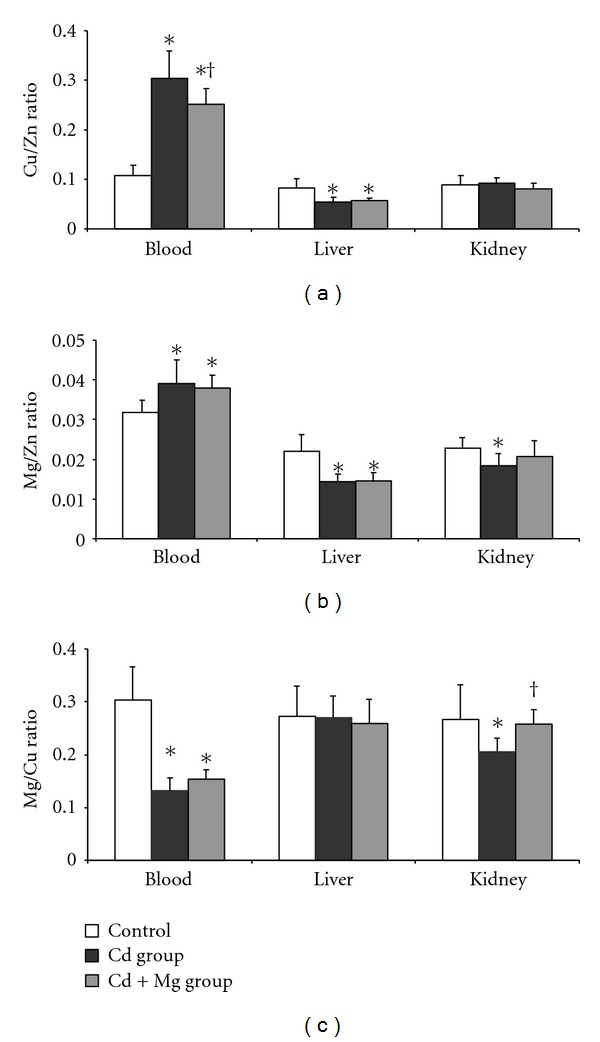
Effect of Cd exposure and Mg supplementation on Cu/Zn ratio (a), Mg/Zn ratio (b), and Mg/Cu ratio (c) in blood, liver, and kidney of rabbits. Control group: nontreated animals. Cd group intoxicated orally for 4 weeks with 10 mg Cd/kg b.w./day. Cd + Mg group given 40 mg Mg/kg b.w. 1 h after Cd treatment. Marked values differ significantly (ANOVA + LSD test) from *control and ^†^Cd group (*P* < 0.05).

Bioelements ratios Cu/Zn, Mg/Zn, and Mg/Cu were calculated for blood, liver, and kidney. Exposure to Cd elevated Cu/Zn ratio in blood but decreased this ratio in liver, while Mg cotreatment markedly reduced this effect of Cd in blood (Figure 2(a)). Similarly, Mg/Zn ratio was significantly increased in blood and decreased in liver and kidney of rabbits intoxicated with Cd. Magnesium treatment did not have any influence on this ratio in blood and liver, but had beneficial effect in kidney where no difference in this ratio between Cd + Mg group and controls was observed (Figure 2(b)). Mg/Cu ratio was reduced in blood and kidney, while no changes were observed in liver of rabbits given Cd only. Magnesium supplementation completely diminished Cd effect on Mg/Cu ratio in kidney returning it to control levels (Figure 2(c)).

Moreover, Pearson's analysis (Table 3) showed a positive correlation between kidney Mg and Cu levels (*r* = 0.789, *P* < 0.05) and kidney Mg and blood Zn concentrations (*r* = 0.719, *P* < 0.05) in Cd group. In Cd + Mg group, positive correlation was observed between Mg and Zn (*r* = 0.685, *P* < 0.05), Mg and Cu in liver (*r* = 0.671, *P* < 0.05), and Mg and Zn in blood (*r* = 0.712, *P* < 0.01).

## 4. Discussion

Having in mind that Cd intoxication induces disbalance of bioelements and that experimental studies proved that magnesium supplementation has beneficial effect on Cd concentration and on some Cd-induced toxic effects [15, 20–24], the question remains whether and how supplemental Mg affects Cd-induced alterations in bioelements status. The results of this study show that cotreatment with Mg in rabbits exposed to prolonged Cd intoxication has at least partly beneficial effect on bioelements Zn, Cu, and Mg in biological fluids, blood and urine, and investigated organs.

The results obtained for Zn indicate that Mg cotreatment manifested positive effect on Cd-induced reduction of Zn blood concentration on the 18th day of experiment. In addition, a positive correlation between Mg and Zn in blood of Cd + Mg group was obtained at the end of the experiment. This effect of Mg could be explained by competitive antagonism between Cd and Mg at the level of GIT, as well as by corrective effect of Mg on Cd-induced extensive elimination of Zn via urine. Extensive loss of Zn via urine as a consequence of Cd exposure was previously confirmed not only in experimental conditions [25, 26], but also in workers exposed to Cd [27]. Moreover, a positive correlation between Cd and Zn in urine was found in individuals exposed to low levels of Cd in the environment [28]. In this study, Mg administration had protective effect on Zn elimination via urine and kept it in the range of control levels. Since it is known that Mg is freely filtered by glomeruli and reabsorbed for almost 90% via paracellular transport and in less extent (10%) by active transport, using TRPM6 channel [29, 30], it could be postulated that interactions between Mg and Zn could take place, at least partly, on glomerular filtrate level or on the level of reabsorption by paracellular route in the proximal tubule and in the thick ascending limb of Henle's loop. Concerning their active transport, it is known that Zn uses different transporters such as ZnT and ZIP transporters which are highly specific [31] and are not proved to be influenced by Mg. Divalent cation channel TRPM7 (transient receptor potential melastatin-related 7), which has very high affinity for Ca and Mg, is also implicated in Zn, as well as in Cd trafficking [32, 33]. However, question remains to what extent Zn can use these channels in conditions of Mg supplementation having in mind that they are strongly downregulated by intracellular levels of Mg^2+^, MgATP, and other Mg nucleotides [33]. The fact that Mg is applied as Mg acetate should be also taken into consideration since TRPM7 activity has been shown to be enhanced by acidic pH [30, 34].

Cadmium caused increase of Zn concentration in liver and spleen and decrease in bone, which is in accordance with reports given for experimental animals as well as for humans [14]. Nevertheless, supplementation with Mg did not modify Zn concentration in all investigated tissues if compared with animals that received Cd. In both Cd and Cd + Mg groups, rise of Zn for more than 60% had occurred in liver where metallothioneins (MTs) are strongly induced by Cd. As Mg is not supposed to be either inductor of MT synthesis or to form complex with MT *in vivo*, it is most likely that Mg has no influence on the accumulation of Zn in liver. Supplementation with Mg even induced significant increase of pancreatic Zn if compared with Cd group and with controls suggesting that Mg favours Zn transfer into the pancreas.

It is well established that Cd has potent influence on Cu body status in spite of stable Cu homeostasis [35, 36]. In this investigation, Cd intoxication induced significant increase of Cu in blood, provoked intensive urinary elimination of Cu, and elevated Cu concentration in kidney, muscle, pancreas, and spleen.

However, Mg supplementation had profound effect on Cu status in Cd-exposed animals. Apart from blood in which no alterations of Cu concentration between Cd and Cd + Mg group were observed, Mg supplementation reduced urinary Cu levels as well as Cu concentration in kidney, skeletal muscle, pancreas, and spleen in which a significant elevation of Cu in rabbits given only Cd was observed. No effect of Mg on Cu concentration in blood could be explained by the fact that Mg probably has no influence on Cu absorption in GIT via MT and DMT1 transporters (divalent metal transporter 1) or Cu transporter CTR1. Supplementation with Mg reduced urinary Cu concentration if compared with animals intoxicated with Cd and resulted in Cu urine concentrations similar to control ones. However, Mg diminished accumulation of Cu that was observed in kidney, skeletal muscle, pancreas, and spleen of Cd group. This contradictory finding could be explained by increased biliary excretion of Cu favored by Mg. There is probably more than one factor that contributes to the biliary excretion of copper. As a cation essential for biosynthesis of energy-reach proteins, Mg is required for the functioning of ATP7B (ATPase copper-transporting polypeptide)—an ATP-dependent copper efflux transporter that is responsible for the canalicular excretion of copper into bile [37]. The other explanation could be connected with the fact that intracellular GSH plays important role in canalicular transport of copper since Mg is a cofactor of enzymes which are involved in synthesis of this important cellular antioxidant. In our previous results, Mg supplementation induced significant increase of GSH content in kidney of mice exposed to subacute Cd intoxication, and this was explained by stimulative effect of Mg on *de novo* synthesis of GSH [20].

Magnesium balance in organism is tightly controlled by the dynamic action of intestinal absorption, exchange with bone, and renal excretion. In our experiment, Cd intoxication reduced blood Mg for more than 20% and induced more than two-fold increase of Mg concentration in urine. This finding is in accordance with our previous and other authors' results [25, 38]. On the other hand, application of Mg provided the maintenance of Mg level in blood and in muscle and spleen (organs in which Mg concentrations were affected by Cd intoxication) in the range of control. Mg elimination via urine was increased throughout the study period if compared to controls, while no significant difference of Mg concentration in urine between animals given Cd and animals cotreated with Mg was observed after 25th and 28th days. This result confirms profound effect of Cd on urinary Mg loss, probably as the result of toxic effect of Cd on epithelium of proximal tubules, sites of Mg reabsorption from primary urine. To explain this effect, metal transporters as well as disturbed junctions between epithelial cells should be taken into consideration.

Cadmium induced significant decrease of Mg concentration in muscle and increase in spleen, while Mg cotreatment entirely prevented these effects of Cd. Unexpected Cd-induced increase of Mg content in spleen could be explained by upstream regulation of TRMP carriers as a result of defense system activation that led to enhanced Mg entrance into spleen cells [39].

In order to give more information on the effect of the enhanced Mg intake on Zn, Cu, and Mg fate in organism we calculated the ratio of Cu/Zn, Mg/Cu, and Mg/Zn concentrations in blood, liver, and kidney. Although insufficient, literature data indicate that ratios of bioelements can provide more realistic view of changes in their concentration in organism than the individual levels of trace elements [40]. To our knowledge, there are no data on the effect of Mg supplementation on Zn, Cu, and Mg ratio during oral exposure to Cd. In this experiment, Cu/Zn ratios in blood of Cd and Cd + Mg group were higher than one obtained in control group. However, Cu/Zn ratio in Cd + Mg group was significantly lower than in Cd group suggesting beneficial effect of Mg supplementation on Zn and Cu distribution in blood. Positive effect of Mg supplementation was also observed in kidney where Mg/Cu ratio was lower in intoxicated animals, but in the range of controls when Mg was applied. Although there was no significant change in Mg or Zn concentration in kidney, Mg/Zn ratio in kidney of Cd group was lower than in controls, and this change was also prevented by Mg treatment. Kidney is regarded as a target organ of Cd toxicity; hence, the fact that Mg showed the most pronounced beneficial effect on bioelements ratio in this organ is of particular importance.

The observed beneficial effect of Mg in rabbits exposed to prolonged Cd intoxication could be explained by direct effect of Mg on Cd concentration in organism, since our previously published results show that Mg supplementation decreases Cd concentration in kidney, spleen, and bones of rabbits exposed to 10 mg Cd/kg b.w. for four weeks [15]. Similarly, we determined positive effect of Mg cotreatment on Cd concentration in kidney, lungs, testes, and spleen of mice [21, 22]. Protective effect of Mg on Cd-induced disbalance of bioelements could be also the result of Mg interactions with Zn and Cu. Although mechanisms of these interactions have not been clarified yet, literature data indicate the complexity of interactions between Mg, Cu, and Zn [22, 41].

## 5. Conclusion and Outlook

This work contributes to investigations on interactions between toxic Cd and bioelements Zn, Cu, and Mg and gives better insight into complex changes that Cd induces in organism. In order to explain the interactions between toxic metal Cd on one side and bioelements Zn, Cu, and Mg on the other, it is essential to clarify the mechanisms of their interactions on the levels of absorption, distribution, and elimination. Furthermore, in order to predict the effect of supplemental Mg on Cd-induced disbalance of bioelements homeostasis, the comprehensive knowledge of the precise molecular mechanisms of Cd biological effect on bioelements status is a necessary step. Thus, better insight into Cd and bioelements transport across cellular membranes is needed. Despite intensive investigations over the last decades, and accumulating evidence for the existence of several metal transporters in mammalian cells, numerous questions, especially those concerning their selectivity and potential similarity between bioelements homeostasis and Cd toxicity, remain to be answered.

This study also contributes to the unsolved problem of prevention and/or therapy of Cd toxicity. Presented findings provide evidence that Mg besides its ability to reduce Cd body burden during intoxication can also prevent, at least partly, Cd-induced alterations in the concentrations of bioelements Zn, Cu, and Mg.

## Figures and Tables

**Table 1:** The effect of Mg supplementation on Zn, Cu, and Mg concentration in the urine of rabbits intoxicated with Cd.

				Days					
	0	10	15	17	19	21	23	25	28
				*Zinc* (*μ*mol/L)					

Cd group^(1)^	9.59 ± 5.14	13.78 ± 9.25	13.17 ± 6.13	17.65 ± 13.15	14.62 ± 7.95	24.20 ± 13.99*	23.00 ± 14.89*	14.18 ± 7.79	18.28 ± 14.33
Cd + Mg group^(2)^	10.69 ± 2.90	10.75 ± 5.78	16.81 ± 8.88	15.04 ± 9.26	14.19 ± 5.73	16.03 ± 8.83	15.73 ± 3.37	10.71 ± 4.76	15.69 ± 6.41

				*Copper* (*μ*mol/L)					

Cd group	0.87 ± 0.37	1.30 ± 0.85	1.48 ± 0.80	1.34 ± 1.24	1.54 ± 0.71*	2.16 ± 1.07**	1.86 ± 1.15*	1.74 ± 1.12*	1.68 ± 0.96*
Cd + Mg group	0.89 ± 0.55	1.19 ± 0.63	1.14 ± 0.80	1.21 ± 1.00	1.10 ± 0.69	0.99 ± 0.60^†^	1.08 ± 0.32	1.12 ± 0.41	1.22 ± 0.52

				*Magnesium* (mmol/L)					

Cd group	22.56 ± 5.96	33.06 ± 21.43	34.94 ± 18.71	43.71 ± 11.38**	36.55 ± 13.75*	44.65 ± 16.89**	44.53 ± 15.48**	51.68 ± 28.07*	48.95 ± 24.42*
Cd + Mg group	23.59 ± 5.70	54.14 ± 26.49^∗∗∗†^	60.96 ± 25.44^∗∗∗†^	74.85 ± 22.37^∗∗∗††^	77.75 ± 27.61^∗∗∗†††^	64.66 ± 16.75^∗∗∗†^	69.40 ± 7.72^∗∗∗††^	66.11 ± 25.54***	60.98 ± 27.92***

^(1)^Cd group intoxicated orally every day for 4 weeks with 10 mg Cd/kg b.w.

^(2)^Cd + Mg group given 40 mg Mg/kg b.w. 1 h after Cd treatment.

Values are presented as the means ± SD. Marked values differ significantly (ANOVA + LSD test) from *control (day 0) and ^†^Cd group.

^∗, †^ *P* < 0.05; ^∗∗, ††^ *P* < 0.01; ^∗∗∗, †††^ *P* < 0.001.

**Table 2:** The effect of Mg supplementation on Zn, Cu, and Mg concentration in investigated tissues of rabbits intoxicated with Cd.

	Zinc concentration (*μ*mol/kg)			Copper concentration (*μ*mol/kg)			Magnesium concentration (mmol/kg)		
	Controls^(1)^	Cd group^(2)^	Cd + Mg group^(3)^	Controls	Cd group	Cd + Mg group	Controls	Cd group	Cd + Mg group
Heart	411.68 ± 50.53	490.41 ± 53.93	439.01 ± 54.03	69.94 ± 5.38	72.90 ± 3.33	69.90 ± 5.03	14.25 ± 1.70	14.55 ± 1.04	14.52 ± 2.43
Lung	438.57 ± 50.93	476.91 ± 50.28	478.29 ± 36.59	27.45 ± 3.90	32.69 ± 3.92	31.94 ± 7.73	12.73 ± 2.15	12.91 ± 2.36	13.44 ± 2.67
Liver	640.73 ± 112.27	1037.02 ± 128.61**	1023.23 ± 178.61**	52.11 ± 9.64	55.55 ± 9.26	58.32 ± 13.09	14.37 ± 1.91	14.94 ± 1.94	14.63 ± 1.50
Kidney	677.15 ± 106.09	825.39 ± 178.96	717.80 ± 80.52	55.07 ± 8.58	74.88 ± 11.37*	57.05 ± 7.63^†††^	14.19 ± 2.87	14.99 ± 3.12	14.62 ± 1.80
Skeletal muscle	202.36 ± 29.29	233.92 ± 52.16	212.12 ± 44.95	12.23 ± 1.54	16.03 ± 1.83**	13.65 ± 2.45^†^	25.84 ± 2.12	21.48 ± 2.44*	23.44 ± 3.73
Brain	255.10 ± 44.35	284.15 ± 15.06	248.82 ± 49.49	37.83 ± 2.92	40.86 ± 3.57	35.03 ± 5.42^††^	10.57 ± 1.30	10.94 ± 1.56	9.17 ± 1.43^†^
Pancreas	649.93 ± 114.89	797.36 ± 49.89	844.37 ± 211.05*	15.05 ± 2.81	19.34 ± 3.68*	16.50 ± 3.02	16.28 ± 1.52	17.56 ± 1.56	19.10 ± 3.47
Spleen	435.44 ± 91.35	591.04 ± 103.48**	536.32 ± 73.90*	32.53 ± 3.87	38.08 ± 4.23*	34.15 ± 2.68	16.59 ± 1.97	21.08 ± 3.68*	17.14 ± 3.64^†^
Bone	3.75 ± 0.42^(a)^	2.61 ± 0.32^∗(a)^	2.55 ± 0.35^∗(a)^	199.65 ± 34.67	201.74 ± 40.27	190.71 ± 26.44	343.46 ± 22.31	340.34 ± 20.41	341.94 ± 25.43

^(1)^Controls—nontreated animals.

^(2)^Cd group intoxicated orally every day for 4 weeks with 10 mg Cd/kg b.w.

^(3)^Cd + Mg group given 40 mg Mg/kg b.w. 1 h after Cd treatment.

Values are presented as the means ± SD. Marked values differ significantly (ANOVA + LSD test) from *control group and ^†^Cd group.

^∗, †^ *P* < 0.05; ^∗∗, ††^ *P* < 0.01; ^∗∗, †††^ *P* < 0.001.

^(a)^mmol/kg.

**Table 3:** Correlation coefficients between the chosen indices of the body status of Zn, Cu, and Mg in animals treated *per os* with 10 mg Cd/kg b.w. and supplemented with 40 mg Mg/kg b.w. daily for 4 weeks.

	Cd group^(1)^			Cd + Mg group^(2)^		
	Mg blood	Mg liver	Mg kidney	Mg blood	Mg liver	Mg kidney
Zn blood	0.210	−0.137	0.719*	0.712*	−0.146	−0.124
Cu blood	−0.159	−0.222	0.166	0.098	0.412	−0.074
Zn liver	0.527	0.492	0.392	−0.218	0.685*	0.320
Cu liver	−0.132	0.340	0.511	−0.269	0.671*	0.200
Zn kidney	−0.322	−0.449	0.592	0.167	−0.061	0.045
Cu kidney	−0.406	−0.422	0.789*	−0.037	−0.009	0.662^§^

^(1)^Cd group intoxicated orally every day for 4 weeks with 10 mg Cd/kg b.w.

^(2)^Cd + Mg group given 40 mg Mg/kg b.w. 1 h after Cd treatment.

Pearson correlation (*r*, *p*); *P < 0.05; ^§^ *P* < 0.06  (*N* = 8).
